# Protecting unaccompanied migrant youth in transit through Mexico: frontline perspectives on appropriate policy implementation

**DOI:** 10.1093/heapol/czaf037

**Published:** 2025-06-05

**Authors:** Susanna Corona Maioli, Rochelle A Burgess

**Affiliations:** UCL Institute for Global Health, 30 Guilford Street, London WC1N 1EH, United Kingdom; UCL Institute for Global Health, 30 Guilford Street, London WC1N 1EH, United Kingdom

**Keywords:** migration, unaccompanied youth, child protection, policy implementation, Mexico

## Abstract

Mexico is the world’s largest global migration corridor and in the last decade there has been an increase in forced migration of families, women, and unaccompanied children and adolescents. The latter population requires specific policy and implementation frameworks due to increased vulnerability related to their age, gender, and unaccompanied status, which can seriously impact their long-term health and wellbeing. However, globally and in Mexico there are reports of lack of appropriate implementation of protection measures. Thus, this article aims to explore the perspectives of frontline workers who conduct daily work with unaccompanied migrant youth. Through 29 semi-structured interviews conducted with different migration workers, mostly based in Mexico, in summer 2021, we found that possibilities for advancing child rights exist even with a scarcity of resources. In fact, although workers highlighted a context of lack of resources, partly determined by a political vision that does not recognize humanity as a priority, specific skills and knowledge were identified for fostering migrant youth resilience. Knowledge such as clear best interests of the child guidelines, and skills such as empathy, adaptation, and recognition of agency of young people enabled some workers to provide appropriate safeguarding. However, we highlight how the contextual scarcity of services overruns individual worker capacities, leading to a lack of appropriate safeguarding overall. Based on worker perspectives, we provide recommendations for appropriate policy implementation. Results are reported according to Consolidated Criteria for Reporting Qualitative Research guidelines.

Key messages

- Despite growing migration of unaccompanied children and adolescents in Mexico, policy implementation of protection measures for this population remains insufficient.
- Interviewed frontline migration workers identified specific skills and knowledge relevant for work with this population, including ways to work regardless of scarcity of resources.
- Individual efforts cannot compensate for lack of regional coordination and funding, highlighting the importance of constant grassroots political advocacy and networking for policy implementation.

## Introduction

Mexico is currently the world’s largest global migration corridor (World Migration Report 2024). In the last decade, there has been an increase in migration of families, women, and unaccompanied children and adolescents in transit through Mexico (SEGOB 2016, UNHCR 2020). The latter come mostly from Guatemala, Honduras, and El Salvador (ibid) and constitute a separate migration category in terms of management due to their extrinsic vulnerability (Corona Maioli *et al*. 2021). Being underage without the care of direct parental guardians predisposes them to become victims of trafficking (CNDH 2009) or to miss out on important formative opportunities (Corona Maioli *et al*. 2024). Despite this, it has been documented that there is a lack of protection for unaccompanied migrant youth in Mexico: of 98 000 underage migrants detained between January 2021 and May 2022, only 19% were processed by the Mexican Child Protection Authority, and of these 55% were deported through ‘collective’, rather than case by case, analysis (WOLA 2023). This is in line with reports from other countries, where there is either a gap related to services specific to this population (Fili and Xythali 2017), a lack of coordination between existing services (Corona Maioli *et al*. 2023), or challenges with existing policy implementation (Lidén *et al*. 2017). This may be partly due to the complexity of addressing the needs of migrant children within a national child welfare system—considered more appropriate than parallel management (Corona Maioli *et al*. 2021)—and partly due to political prioritization of objectives that are opposite to those in the best interest of the child (Barbulescu and Grugel 2016).

### Working with unaccompanied migrant youth in complex policy landscapes

The social environment migrant youth face, relevant for their health and wellbeing, is highly dependent on the mediation of professionals who interact with them, guiding them through regularization or access to health services. Many young people have hostile encounters with authorities, with reports of abuse and violence (Sawyer and Márquez 2017). This creates complex situations to address since practitioners need to deal with mistrust of migrant children or adolescents, often resulting in impatience at long waiting times for their regularization, and escapes from shelters (Clayton et al. 2019).

Research on perspectives of carers and professionals working with unaccompanied migrant youth has highlighted several challenges for these professionals. For example, studies of mental health service utilization for unaccompanied refugee minors in the UK and of long-term foster care in the USA identified language and cultural barriers (Crea *et al*. 2018, Majumder *et al*. 2019). Therapists’ gender was considered a potential cultural issue, and therapeutic approaches applied by Western therapists (e.g. talking) were not necessarily understood or valued in the same way by refugee youth (ibid). Especially in precarious situations, many prefer practical, future-oriented solutions rather than remembering traumatic experiences. A Spanish study exploring perspectives of workers in the education system described a lack of specific training for the inclusion of migrant minors, as well as the conflict between working and studying among unaccompanied migrant youth (Nublado Menaya 2020). In fact, many travel to support their families or carry the burden of debt, prioritizing work over education (Corona-Maioli *et al*. 2023). In the USA, a lack of clarity over specific procedures for unaccompanied youth was highlighted in a study looking into social worker perspectives: findings note that the complex and different legal routes that an unaccompanied minor can undertake, sometimes simultaneously, greatly influence both the assistance that can be given and the mental health of the young person (Clements et al. 2020).

In addition to these challenges, working as a frontline migration worker has become increasingly politicized and complex (Humphris and Sigona 2021), to the point of threatening personal security (Tondo 2024). Within this context this study aimed to explore frontline worker perspectives of dealing with migration of unaccompanied migrant children and adolescents in Mexico, complementing existing studies (Sánchez and Polanco-Hernández 2024). The study explores the following research question.

- How do practitioners perceive the ability of policies and implementation in Mexico to meet the needs of unaccompanied young migrants?

### Guidelines on appropriate care for migrant children and adolescents

Appropriate care for unaccompanied children and adolescents should be understood as a series of specific phases: detection, first reception, longer-term care, and transition to independent living (UNICEF, DIF 2019). Regarding detection, migration officers should be trained and one of these trained officers should always be available as a direct reference to ensure an identified or suspected unaccompanied child or adolescent is swiftly referred to appropriate first-reception centres (ideally within 24 h). Following detection, there should also be identification of specific immediate needs in a child-friendly way, such as if the child could be a victim of trafficking, if there is a disability, a pregnancy, or another immediate health need. In principle, children and adolescents should never be kept in detention (UNHCR 1997, EASO 2019, UNICEF, DIF 2019), since detention is considered always a violation of the best interests of the child. Mexican law (described subsequently) also ratifies the principle of non-refoulement, so by law children and adolescents cannot be deported without due process. The EU Agency for Asylum (previously EASO, now EUAA) guidelines on Best Interests of the Child (BIC) procedures (ibid) also state that children should be exempted from accelerated procedures at the border when adequate support cannot be secured (exceptions that may determine an accelerated border procedure include: the unaccompanied child asylum applicant comes from a country of origin considered safe, the applicant is considered a danger to national security or public order, the applicant introduced a subsequent application which is not admissible, or the applicant presented false documents or destroyed an identity or travel document). Thus, children must always have access to legal advice free of charge from this very first phase and be informed of their process in a language they can understand, if necessary with a mediator or translator.

In first reception, siblings should be kept together, and this stage should not last more than 45 days (UNHCR 1997, UNICEF DIF 2019). During this time, a multidisciplinary team outlines a durable solution in accordance with the best interests of the child principle (the best interests of the child principle is ensuring the full and effective enjoyment of all the rights recognized in the Convention of the Rights of the Child and the holistic development of the child. This goes beyond safety and protection and includes non-discrimination, wellbeing, family reunification if within the child’s wishes and best interests, and access to health and education, among other aspects.) (EASO 2019). It is usually the Child Protection Authority, such as in Mexico, or a Family Welfare Institution, such as in Colombia, that is responsible for identifying a rights and protection restitution plan. Individualization of this plan occurs through a series of child-friendly interviews and following the assignment of a guardian or individual reference operator who is able to guide the child or adolescent into a specific, case-by-case process (UNHCR 1997). During the first-reception phase there is guided access to health, mental health, and education services, as well as regular legal advice to assist in longer migration-regularization processes, such as an asylum request. If the child’s process results in the need for longer-term care, family-based solutions should be prioritized. This requires that institutional operators must find out about the child’s family circumstances, including extended family, and complete family tracing or otherwise explore options for a foster family if in agreement with the child’s preferences (Corona-Maioli *et al*. 2023).

The longer-term care phase can be considered as a bridge to transition to complete independent living. Providing for living arrangements in longer-term care may include residential care or supervised independent-living arrangements (UNHCR 2021). In addition to continuing guidance on the individualized life plan, care should also include referrals for the child or adolescent to community integration activities such as sport or cultural activities. In this stage, periodic monitoring and validation of the protection measures and of the individualized life plan established at the initial reception are also required (UNHCR 1997, EASO 2019). The final outcome may result in a return to country of origin, reunification with family in a different country, or settlement into the country of first reception. Ideally, there should be an ‘after-care’ service also in this last phase, in which the adolescent can maintain contact with a reference person for follow-up, advice, and support.

### Legislative and institutional framework of care of migrant children in Mexico

In principle, Mexico has a good legal and institutional structure to protect unaccompanied migrant children. The legal basis can be conceptualized on the following three levels (Bucknall *et al*. 2019). First, the Mexican Constitution (1824), which grants all people on Mexican territory human rights including right to education, health, and shelter. Second, the Migration Law (2011), which extends these rights including the right to due process on Mexican territory and the conceptualization of irregular entry as an administrative, not criminal, fault. Third, the Law on Refugees and Complementary Protection (2011), which states the right of any non-Mexican to seek asylum in Mexico on the basis of the 1951 Refugee Convention and the extended definition of the Cartagena Declaration (1984). The basis for obtaining refugee status according to the 1951 Refugee Convention and its 1967 Protocol is: credible fear of persecution in country of origin ‘for reasons of race, religion, nationality, membership of a particular social group, or political opinion’. The Cartagena Declaration expands the refugee definition to include threats to life, safety, or freedom ‘by generalized violence, foreign aggression, internal conflicts, massive violations of human rights, or other circumstances’. Mexico also has a specific law for the protection of children, called Ley General de los Derechos de Niños, Niñas y Adolescentes (LGDNNA 2014) which explicitly mentions rights of migrant children (pp. 39) in line with the United Nations Convention on the Rights of the Child (1989).

The LGDNNA clarifies an institutional structure responsible for management and protection of unaccompanied migrant children (Table 1). The institutions involved are the Instituto Nacional de Migración (INAMI) and Comisión Mexicana de Ayuda a Refugiados (COMAR) regarding detection and eventual regularization as a refugee, and the Child Protection Authority (Procuraduría de Protección de Niños, Niñas y Adolescentes or PPNNA) and Child Welfare (Sistema Nacional de Desarrollo Integral de la Familia or SNDIF) regarding child protection. Once an unaccompanied child is identified by INAMI, he or she is transferred to a SNDIF for their shelter and safeguarding, with guardianship under the PPNNA. Thus, PPNNA must be notified (also if an unaccompanied child arrives at a non-governmental migrant shelter) and it is only PPNNA that can determine protection measures in the best interests of the child: family reunification, return, asylum request, or other migration regularization to be done in partnership with INAMI and COMAR.

**Table 1.** Institutional structure of unaccompanied child protection in Mexico.

Institution	Role
Instituto Nacional de Migración (through child protection officers or Oficial de Protección a la Infancia)	First detection of an unaccompanied non-Mexican child or adolescent and immediate transfer to a SNDIF
Comisión Mexicana de Ayuda a Refugiados	In the case of non-Mexican unaccompanied children, determination of their asylum request if this is within their will and in their best interest
Sistema Nacional de Desarrollo Integral de la Familia	Child welfare: shelter, safeguarding, educational, recreative, or other activities depending on the capacity of individual SNDIF
Procuraduría de Protección de Niños, Niñas y Adolescentes	Legal guardianship of all unaccompanied children and determination of protection measures based on their best interest

It is worth mentioning that both COMAR and PPNNA report a severe lack of funding in relation to the vastness of their work (with all refugees and all children, respectively) (Bucknall *et al*. 2019, Xantomila 2022). In addition, SNDIF shelters are not only for unaccompanied migrant children, who often have specific processes and require specific training of staff. Thus, although the LGDNNA states the right of migrant children to be duly informed and have legal representation, often this occurs only if they are transferred by PPNNA to a non-governmental shelter (due to lack of space in SNDIF) or after their arrival in these spaces on their own (Bucknall et al. 2019). Most cases, as reported above, result in deportations (Amnesty International 2021).

## Materials and methods

### Research context

This research was conducted as part of a broader project exploring the impact of migration transit on the psychosocial mental health of unaccompanied youth in Mexico. A total of 18 migrant adolescents and 29 workers with jobs that related directly or indirectly to unaccompanied youth migration were interviewed by the first author in two migrant shelters in Mexico City and Guadalajara, in June and July 2021. Details and results of the interviews with migrant adolescents are reported elsewhere (Corona Maioli et al. 2024). Workers were recruited purposively either on site at the shelters or by email contact and snowball sampling (Table 2). They were interviewed in person or online, depending on preference. The context at the time of the research was the ongoing Covid-19 pandemic, which resulted in fewer people being hosted in shelters for long periods of time. Strict safety measures were implemented during shelter visits (face mask, hand washing, testing, and isolation from other social contact).

**Table 2.** Worker participants interviewed.

Role	Participants
Shelter psychologist/psychiatrist	3
Shelter lawyer	2
Migration academic	3
Shelter or NGO coordinator	5
Shelter volunteer	2
Shelter sister	1
Shelter staff	1
Shelter director	4
Child Protection Authority (Procuraduría) Officers	2
Shelter or NGO social worker	2
UN worker	2
National Institute of Migration (INM) Child Protection Officer	1
UN Child Protection Officer	1

### Methods

The methods used were qualitative semi-structured interviews, lasting 30–40 min on average, recorded and transcribed in Spanish. Interviews were first coded aided by the contextualization of 2 months of ethnographic observation by the first author in the shelters, with ethnographic notes coded accordingly. Data analysis was conducted by reflexive thematic analysis following the steps outlined by Braun and Clarke (Braun and Clarke 2006). Namely, codes were organized into overarching themes that enabled common patterns in the dataset, which were then organized into thematic categories that allowed clear argumentative claims to be made about the data (Burgess *et al.* 2022). Two independent coders triangulated the analysis of 20 interviews.

## Results

We report on two thematic categories derived from workers’ interviews, which describe the nature of their daily work with unaccompanied migrant youth within extremely restricted governmental services and resources. Thus, it can be framed as an analysis of how this work was done regardless of the lack of implementation of the adequate policy frameworks that are in place. On one hand, workers described a context of extreme precarity: few and underfunded services and nonexistent mental health support—described in the first thematic category ‘unmet need on behalf of authorities and child protection’. On the other hand, the thematic category of ‘acompañamiento*’* (‘representativeness’) captures through professionals’ accounts a compensation, or reaction, to this context: a description of how they advanced understandings of migrant child rights.

### Unmet need on behalf of authorities and child protection

Professionals highlighted how the intersection of different needs, from basic food and shelter to work and higher education, combined with restrictive migration policies and social discrimination created difficult and complex situations to address. Specifically, workers emphasized lack of funding for the institutions meant to protect migrant children, leading to lack of infrastructure and staff:

‘Here in Jalisco we do not have a State shelter for migrant adolescents, so we look for alliances with civil society’ (Child Protection Authority Officer)

This means that there is insufficient support for clear and established institutional pathways that all migrant adolescents can follow to obtain shelter and regularization in Mexico. As a migration academic says, this means many migrant adolescents ‘*are not going to school, are not receiving any follow up, medical checkup nor an appropriate nutrition*’. Thus, as the Child Protection Authority Officer mentioned, civil society compensates with a patchy national distribution:

‘We have to divide ourselves in a million pieces to meet all the demands (…) we know we are not specialized [in child migration], but there is no one else who can do this’ (Shelter coordinator)

Necessary resources were categorized into three subthemes: ‘capacity’, i.e. physical spaces to receive migrant youth; ‘continuity’, i.e. enough trained staff to ensure appropriate follow-up; and ‘coordination’ between different institutions and Mexican states. This latter resource was identified as important in order to not re-victimize, as emphasized in a systematic review of facilitators for disclosure of refugee children life stories (van Os *et al.* 2020).

Finally, workers placed the scarcity of services within a context of adverse social determinants of health, including difficult access to health services and lack of mental health services for adolescents. As a shelter psychiatrist says in relation to mental health services for migrant adolescents:

‘Well, they don’t exist [even] for Mexicans. [I work in] a third-level hospital where severe and complex cases are taken to, but [what is missing] is primary care. There is no prevention, including social determinants of health’ (Shelter psychiatrist)

### Acompañamiento

As stated in different guidelines, such as those of the United Nations Children’s Fund (UNICEF) and DIF (UNICEF DIF 2019), appropriate care of migrant children—especially if unaccompanied—requires a highly individualized evaluation. The word ‘acompañamiento’, in Spanish, means ‘accompaniment’, which we translate as ‘representativeness’, or acting on behalf of migrant youth in their best interests. As a shelter director explained:

‘I think sometimes to be their voice, to be present, to represent them… I think that is what right now we have been able to do for them [migrant youth]*’* (Shelter director)

This thematic category captured skills and knowledge identified among professionals as necessary for this work (Table 3). Here we define ‘skills’ as practical-interactional expertise, and ‘knowledge’ as theoretical (then applied) expertise.

**Table 3.** Skills and knowledge identified by workers as relevant for work with unaccompanied migrant youth.

Skills	Knowledge
Responsibility Empathy Listening ability Adaptation Recognition	Of migration context Of interview techniques Of best interests of the child Of advocacy techniques

Among skills identified as relevant, adaptation is required because migration dynamics change constantly:

‘We keep changing the care model, because a lot depends on circumstances of changing profiles or situations’ (Shelter director)

‘It is difficult because we can’t have a standard procedure that we can repeat’ (Shelter coordinator)

A sense of responsibility and empathy form the basis for the gratification of working with migrant youth, sometimes tiring and frustrating due to the precarious context:

‘I think seeing how these kids, for example through the programmes we have here [in the shelter], someone who came with only third year of primary school suddenly finishes primary school (…) or someone who says they learned bread-making [in the shelter] and found a baker job in the US. That gives satisfaction, it resonates and tells you it is worth it. It is worth it, sometimes even the tiredness, the lack of sleep, sometimes even the anger because one is not made of stone (…) but I think more than those things [difficulties], there is more satisfaction’ (Shelter sister)

Recognition refers to stepping out of personal biases and acknowledging the agency and humanity of migrant youth as equals, which ‘*creates a space where they can feel they are not being questioned, that their opinion is valid*’ (NGO social worker). This means also stepping out of adult-centred thinking:

‘Sometimes it seems like the young person needs to study (…) because I say so as an adult, or work because that is the need of the family, [but] that may not be what the young person wants in that moment’ (Shelter social worker)

Still, power imbalance and the limits inherent in the specific professional role should be admitted. These skills need to be complemented with up-to-date knowledge of the migration context, of interview and advocacy techniques, and of the best interests of the child (such as rights to legal identity, family reunification, integration, health, and education). Making interviews shorter, carrying out repeat interviews, and establishing rapport, were highlighted as beneficial for interviewing migrant youth:

‘We have to speak [to them] with a communication that is age and context-appropriate (…) [because] if you want to help a person you have to make her first understand [the situation], so s/he participates and recognizes herself as a rights holder’ (NGO lawyer)

## Discussion

This study highlights how the skills practitioners need to work with unaccompanied migrant youth depend on more than just resources, namely on a deep sense of responsibility, empathy, and humanity. It is critical to ensure that these emotional skills are not assumed, but rather embedded into training alongside appropriate knowledge of the context. Civil society workers interviewed in this study worked to inform and guide migrant youth they interacted with, recognizing first their agency and independence in taking their own decisions. This point is relevant, since the initial encounter with an unaccompanied adolescent may determine whether or not he or she would plan to escape subsequently. Other studies have emphasized the value of recognition as useful both for psychological and legal interviews with migrant youth (Ruiz Rosado 2018, Matlow *et al.* 2023): practitioner humility (being self-aware of our own biases and of how little we know about the young person) is the basis for creating trust. Of crucial importance is the ability to listen and to truly understand the will of the unaccompanied child or adolescent, possibly with more than one interview, without imposing an adult-centred version of facts. As mentioned above, practical solutions will often be prioritized over studying or obtaining psychological support.

Workers highlighted the role of empathy in their work, as well as strong responsibility towards younger generations. This is important because it transforms work that may seem administrative into an actual human encounter in which wellbeing is placed at the centre, or the best interests of the child. For example, in typical case-by-case management, assignment of a guardian or at least a reference operator is recommended, as stated in the guidelines for adequate protection of unaccompanied children (UNHCR 1997, UNICEF DIF 2019). These guidelines highlight the importance of access to legal representation and advice in the first-reception phase, as well as placing children into a clear roadmap of protection, from initial reception centres to eventual foster care, family reunification, or supervised independent living arrangements (UNHCR 2021). In Mexican legislation, the concept of ‘best interests of the child’ is explicitly stated but never defined clearly. Our findings stress that empathetic practice is central to the operationalization of this concept.

Enacting these guidelines demands capacity of highly trained staff, which is lacking in the Mexican context and results in mental health strain on aid professionals (Sánchez and Polanco-Hernández 2024). However, lack of overall capacity does not remove importance from any single empathic interaction that may take place: these acts of resistance to a broadly hostile and security-oriented migration system are concrete forms of creating transit routes to safety and forming protective networks for migrant youth. Anthropologist Valentina Glockner noted these acts of resistance at the micro-level in her ethnography of migrant caravans, emphasizing the importance of the help activists gave to an adolescent she encountered in fighting his deportation (Fagetti 2019).

Our work highlights that knowledge of specific geopolitical contexts is essential, regardless of whether the job is not strictly ‘political’ (e.g. like a migration official), which has been supported by evidence in other studies. For example, a scoping review of key competencies in child refugee mental health identified that knowledge of the complexity of migration management, political advocacy skills, culturally appropriate holistic approaches, and regional coordination with others in the child’s network (both institutional and family-wise) were uncommon but essential (Due and Currie 2022). This is challenging, because the migration context changes rapidly and it is full of politically induced dangers, as well as the increasing presence of organized crime (de la Rosa Rodríguez 2022). Thus, we highlight the important skill of adaptation identified by workers, which demands constant rethinking and reworking of current guidelines, as well as tailored implementation. The complexity of migration management manifests on multiple levels: from immediate shelter and safety concerns to medium- and long-term management. As mentioned by workers and in the scoping review, this demands territorial coordination of a network of services, and Mexico actually has an institutional body that is meant to conduct this coordination: the Sistema Nacional de Protección de Niños, Niñas y Adolescentes (SIPINNA), which is currently underfunded. Initial funding to a coordinating body could be useful for prioritization of funds, including support for mental health of migration professionals. Migration management has to be conceptualized as complex and functional in the long-term (Scholten 2020), something that short-term political mandates tend to avoid.

Finally, our work is in agreement with wider evidence suggesting that advocating for humane social representation of migrants is important in shifting perceptions of migration, leveraging political will, and increasing empathy in social interactions (Clayton *et al.* 2019). This reflects the importance of specific training for officials who deal with migrants or children more broadly (such as COMAR or SNDIF workers), who may have biased beliefs about the implications of unaccompanied child migration. In a health promotion framework (Ottawa Health Promotion Conference 1986), any action designed to obtain ‘political commitment, policy support, or social acceptance’ can be defined as advocacy and it can be oriented to the health goal of enabling adequate living environments for migrant youth. Frontline workers are pivotal in mediating this change and in creating these environments in the shelters where they work. Broader advocacy from academia and policy circles is nonetheless necessary, taking into account the high rates of burnout of frontline workers and the monitoring of their own mental health as a central component of migration management. With this in mind, strategies that expand the capacity of migration services are needed: for example, foster families or peer mentorship (Corona Maioli *et al.* 2021, Corona-Maioli *et al.* 2023). Appropriate access to school and education, as well as viable work–study options (Clements *et al.* 2020, Nublado Menaya 2020), can also expand the scope of migration workers to the education system: if done appropriately, this could facilitate integration efforts and enhance interaction with Mexican peers.

## Conclusions and policy recommendations

We presented the perspectives of migration workers who emphasized lack of resources, spaces, and specialized staff to deal with unaccompanied child migration in Mexico, yet also identified specific skills and knowledge necessary for working with migrant children—which can be the basis for future work. Specific policy recommendations we draw are: to define the best interest of the child principle in national legal documents, to assign enough funds firstly to a coordinating and/or monitoring body that can oversee the territorial distribution of services on a national level, to assign an adequate number of Child Protection Officers based on each state’s number of registered children identified by INAMI, to update guidelines and funding every year according to priorities outlined by the coordinating body, to prioritize strategies to increase open-door shelters or family fostering, and to guarantee specific training on unconscious bias and migration knowledge to institutional staff who deal with unaccompanied migrant children.

## Data Availability

The data underlying this article will be shared on reasonable request to the corresponding author.

## Reflexivity statement

S.C.M. is a Mexican–Italian medical doctor with an interest in the impact of forced migration on health, particularly for younger people. Due to her own migratory experience, she believes in the benefits of freedom of movement and conducted this independent project aided by the fact that Spanish is her mother-tongue and she has lived in Mexico previously. R.A.B. is a UK-based scholar and activist who identifies as a migrant of Black Caribbean heritage. Her work on mental health and social justice helps to advocate for improved mental health policy and practice landscapes for marginalized peoples globally. She draws on her familial history of oppression and exclusion to inform her feminist scholar-activism.
